# Birth Paralysis of the Upper Extremity

**Published:** 1903-02-14

**Authors:** 


### BIRTH PARALYSIS OF THE UPPER EXTREMITY.

Birth paralysis affecting the upper extremity is now a well recognised affection which has been traced pretty definitely to injury of the motor nerve fibres which run in the anterior divisions of the fifth and sixth cervical nerves at their point of junction. Dr. Robert Kennedy,[1] Regius Professor of Surgery in the University of Glasgow, who has lately been repairing this injury by the early suture of the brachial plexus, says that in some way, during the birth of the child, tension or compression of the upper part of the brachial plexus has been caused with the result that the nerve fibres of the fifth and sixth nerves have had their conductivity destroyed. Some maintain that the lesion is caused by the compression of the nerves between the clavicle and the transverse processes of the vertebræ, while others think that the damage is caused by the nerves being crushed between the clavicle and the first rib. As a rule, he says, there is the history of considerable force having been applied during the birth of the child. In some cases after the birth of the head the finger of the accoucheur has been hooked into the axilla and considerable traction exerted; while in certain cases of breech presentation, or of transverse presentation in which turning has been performed, the arm has slipped up over the head and has required considerable force to dislodge it; or the body has been at the same time pulled upon in such a way that the head has been extremely rotated and bent to the side. In this way the nerves are overstretched, causing rupture of the nerve fibres or even complete rupture of the nerves themselves. Whatever may be the presentation, Dr. Kennedy is convinced that the chief factor in producing the lesion is forcible depression of the shoulder while the head is bent to the opposite side and rotated. On examination it is found that the arm does not take part in the usual active movements of the child, but hangs helpless. "It cannot be abducted at the shoulder on account of the paralysis of the deltoid and supraspinatus; the forearm cannot be flexed on account of the paralysis of the biceps, brachialis anticus, and supinator longus. The forearm is usually in extreme pronation on account of the usually present paralysis of the supinator brevis, and the entire arm is rotated inwards, so that the palm of the hand may be directed outwards, this being caused by the paralysis of the external rotators of the humerus (infraspinatus and teres minor), the unopposed action of the internal rotators thus bringing about the characteristic position." The treatment usually adopted in such cases is the use of massage, electric currents, and passive movement; and in certain cases the muscles recover. On the other hand the treatment often fails, the restoration being only partial, or no improvement at all taking place. There is no doubt at all that some cases recover without treatment, and it may be questioned whether the above-mentioned routine has much to do with the recoveries which take place under it. Supposing that the nerves are merely over-stretched, possibly with rupture of the nerve fibres within the sheath, the recovery is almost certain, while if the nerve trunks are completely torn across, although complete recovery is possible, recovery may fail completely or only take place partially. Moreover the same force which produced the injury to the nerves may have done other damage causing such cicatrisation in the neighbourhood as to interfere greatly with the prospects of recovery. From these considerations Dr. Kennedy holds that the only rational way to treat such cases of birth paralysis is to deal with them just as injuries to the peripheral nerves in general are dealt with. As there is a possibility of spontaneous recovery taking place it is right to delay operation for a certain time to see if the developing electrical reactions indicate an approaching recovery of the muscles; but when this time has elapsed without sign of improvement there must be no further delay. Dr. Kennedy would not operate before the child is two months old. By that time it is possible to obtain good electrical contractions of the muscles by the faradic current. If these are obtained then the case ought to be left for a further period, and if after the lapse of another month good contractions are obtained with the faradic current in all the affected muscles, then the case may be expected to recover naturally. If, however, by the time the child is two months old no response can be got in the muscles by the faradic current, although of course the galvanic current evokes good contractions, it is safer to proceed with the operation than to put off in the hope that recovery may eventually take place. Dr. Kennedy has operated on three cases: one aged two months, one aged six months, and another, which must be placed in quite a different category, aged fourteen years. In the first infant the result was really good—a cure. In the second infant the result was very promising; while in the youth, as was perhaps to be expected, after so long a time, no benefit seems to have been obtained. Yet even in this most unpromising case there appears to have been some improvement in the electrical reactions, whatever that may presage in the end. The details of the operation for suture of the brachial plexus are interesting, and should be carefully studied by any who propose to undertake the operation. It is evident however that as in other cases of injury to nerves it is well not to delay treatment, and this is a matter to be borne in mind by those who have opportunities for seeing such cases at an early stage.

[1] Brit. Med. Jour., Feb. 7.

